# Relation between carotid vulnerable plaques and peripheral leukocyte: a case-control study of comparison utilizing multi-parametric contrast-enhanced ultrasound

**DOI:** 10.1186/s12880-019-0374-9

**Published:** 2019-08-23

**Authors:** Xianghong Luo, Wanbin Li, Yun Bai, Lianfang Du, Rong Wu, Zhaojun Li

**Affiliations:** 1 Department of Echocardiography, Shanghai General Hospital, Shanghai Jiaotong University School of Medicine, Shanghai, 200080 China; 2 Department of Ultrasound, Shanghai General Hospital, Shanghai Jiaotong University School of Medicine, Shanghai, 200080 China

**Keywords:** Ultrasonography, Cerebral infarction, Carotid artery, Atherosclerosis, Leukocyte

## Abstract

**Background:**

This study evaluates carotid vulnerable plaques using contrast-enhanced ultrasound (CEUS) and explores the relationship between vulnerable plaques and leukocytes.

**Methods:**

Sixty-two symptomatic and 54 asymptomatic patients underwent CEUS. The images were analyzed using time-intensity and fitting curves, and peak (P_TIC_), mean (M_TIC_), peak (P_FC_), sharpness (S_FC_), and area under the curve (AUC_FC_) were obtained. The relations between CEUS parameters and leukocytes were analyzed.

**Results:**

In the symptomatic group, total leukocytes and neutrophils were higher, while lymphocyte was decreased; P_TIC_, M_TIC_, P_FC_, S_FC_, and AUC_FC_ were significantly higher; M_TIC_ and AUC_FC_ were negatively correlated with lymphocytes, and M_TIC_ was positively correlated with neutrophils. Classification and regression tree analysis showed that M_TIC_ at a cutoff of 20.8 and AUC_FC_ at a cutoff of 8.8 resulted in a predictive of acute cerebral infarction, accuracy of 84.3%, sensitivity of 87.1%, and specificity of 81.5%.

**Conclusions:**

The variation in the perivascular leucocyte is significantly related to intraplaque inflammatory activities, CEUS is a feasible monitor of intraplaque neovascularization, so CEUS combined with perivascular leucocyte could be helpful as a warning for vulnerable plaques.

## Background

Acute cerebral infarction (ACI) is the main cause of adult disability, cognitive impairment, and mortality worldwide [1]. The large artery atherosclerosis cerebral infarction subtype is closely correlated with the presence of vulnerable plaques [2]. Intraplaque neovascularization (IPN) is also a surrogate marker of vulnerable plaques. Previous studies have confirmed a pronounced association between IPN, plaque vulnerability, and cerebrovascular events [3]. Plaque rupture and clinical events appear to be initiated and triggered by vascular leakage, inflammatory cell recruitment, and intraplaque hemorrhage, and all of these are consistent with plaque inflammation processes. Inflammatory activity in the plaque is closely associated with the inflammatory state of the outside system. Notably, atherosclerotic lesions, which contain monocyte-derived macrophages, T lymphocytes, and leukocytosis, are involved in plaque thickness in the carotid artery [4]. Variations in the leucocyte count in the peripheral circulating blood can reflect the inflammatory state of the system to some extent [5].

Contrast-enhanced ultrasound (CEUS) provides direct visualization of the IPN in carotid plaques [6]. Several studies have recently described a positive correlation between the histological density of neovessels and the presence of neovascularization in carotid plaques detected by CEUS [7].

Leukocyte count is a common blood test in clinical practice. A high leukocyte count may reflect a chronic inflammatory state and contribute directly to atherosclerosis through specific mechanisms [8]. The peripheral leukocyte count is a non-traditional risk factor of ACI and has predictive value for ACI prognosis [9]. A relationship between plaque angiogenesis and increased plaque instability and ACI has been established; however, the implications of the peripheral leukocyte count and IPN in carotid plaques assessed by CEUS remain unknown. Therefore, this study investigated the relationship between the peripheral leukocyte count and IPN.

## Methods

### Patient selection

We searched the patient database at the Department of Neurology, Shanghai General Hospital Health System, and an institution with Primary Stroke Center Certification from the China Stroke Center Alliance, to identify all patients who underwent CEUS of the carotid arteries between June 2016 and June 2017. The inclusion criteria were ≥ 50% extracranial carotid artery stenosis secondary to atherosclerosis disease based on a conventional carotid ultrasound, CTA, MRA, and/or catheter angiogram. Patients were then classified as symptomatic, defined as having ischemic neurological symptoms (stroke, transient ischemic attack, or amaurosis fugax) relevant to the index carotid lesion within 7 days of undergoing CEUS, or as asymptomatic. Exclusion criteria included (1) non-atherosclerotic intracranial vascular pathology (e.g., reversible cerebral vasoconstriction syndrome, Moyamoya disease, vasculitis), (2) the presence of potential sources of cardioembolism, (3) contradictions for the use of ultrasonic contrast agents, such as acute cardiac failure, unstable angina, and acute endocarditis, (4) known allergy to micro-bubble contrast agents, (5) a recent history of active bleeding, (6) patients with hematological diseases, malignant tumors, or severe liver, kidney, or pulmonary diseases, or (7) patients had underwent endarterectomy previously.

During the study period, 849 patients underwent the ultrasound, CTA, MRA, or catheter angiogram of the carotid arteries; 551 patients were excluded secondary to a lack of significant stenosis (≥50%). One hundred twenty-eight were excluded secondary to conflict causes of neurological symptoms, e.g., lacunar infarction or cardiac emboli. Thirty-five patients were excluded for previous carotid endarterectomy with restenosis and 19 for tandem lesions. Finally, 54 patients classified as asymptomatic had no history of symptoms, neither remote or at the time of examination, as determined with a detailed neurological examination by an experienced vascular surgeon. Sixty-two patients who were included in this study had acute large artery atherosclerotic stroke. To further analyze the impact of the medical history on the results, the patients were divided into two subgroups: Subgroup 1 (with a history of ACI, 28 patients) and Subgroup 2 (ACI for the first time, 34 patients) and were and analyzed using stratified analysis. To prove whether the IPN from CEUS and leukocyte count are related, we compared the data from the ACI patients with the different medical histories.

All subjects stopped daily medications 24 h before the examination and did not take their blood pressure medication on the day of examination. The general information and medical history of each patient were collected. Diabetes mellitus was diagnosed according to the WHO diagnostic criteria, which are random plasma glucose more than 11.1 mmol/L or fasting plasma glucose 7 mmol/L or higher [10]. The diagnostic standard for hypertension was based on the American Heart Association/American College of Cardiology guidelines. The morning fasting venous blood samples of all subjects were collected to analyze the total leukocyte, lymphocyte, and neutrophil counts. The systolic pressure and diastolic pressure of the brachial artery were measured three times in a resting state, which was taken as an average.

Clinical investigations were performed according to the Declaration of Helsinki. The study protocol was approved by the ethics committee of Shanghai General Hospital (2017KY009) and registered with the official website of China Clinical Trial Registration Center (ChiCTR1800016590). The inform consents were signed by all subjects.

### Instruments and methods

Siemens S2000 (Siemens, Berlin, Germany) and Sequoia 512 ultrasound systems (Siemens Berlin, Germany) equipped with a 9-4-L MHz linear transducer and ultrasound contrast software (Cadence Contrast Pulse Sequencing) were used to acquire standard carotid ultrasound including color Doppler and CEUS.

Carotid ultrasound acquisition: All subjects had rested for 10 min in the supine position. According to previous research methods, both the left and right common carotid arteries, internal carotid arteries, external carotid arteries, and vertebral arteries were imaged by standard carotid ultrasound to record the number of atherosclerotic plaques and their distribution and to evaluate the degree of carotid artery stenosis [11, 12]. CEUS was performed on obvious plaques: (1) the far wall of carotid bifurcation or the initial part of the internal carotid artery; (2) those thicker than 2 mm, choosing the largest; (3) no calcification or the least calcified plaque; and (4) cerebral infraction ipsilateral to the side of the carotid plaque. Obvious plaque area (OPA), calcified area (CA), and calcified area/total plaque area (CA/TPA) were measured using velocity vector imaging (Siemens Medical Systems) [13]. TPA was defined as the sum of all plaque areas measured in any of the carotid artery segments within an individual [14, 15]. The contrast mode of the ultrasound system was fixed (presetting): mechanical index of 0.06–0.08, 90% gain, imaging depth of 2–3 cm and focusing on the back of the ideal plaques. SonoVue™ contrast agent (Bracco, Milan, Italy), 2.5 ml, was injected via the elbow vein in a 2.5 ml bolus followed by a saline flush of 5 ml NaCl 0.9% solution. For offline analysis, 90 s of cine clips were continuously digitally stored.

For quantitative analysis of the CEUS, quantitative analysis software (QontraXt, Esaote, Genoa, Italy) was adopted. The analytical method was chosen to acquire time-signal intensity curve quantitative parameters including the peak time-signal intensity (P_TIC_) and the mean time-signal intensity (M_TIC_), and the quantitative parameters for fitting the gamma curve of the TIC (FC) were maximum peak value (P_FC_), sharpness (S_FC_), and area under the curve (AUC_FC_).

### Statistical analysis

Statistical analysis of the data was performed using SPSS version 13.0 software for Windows (SPSS Inc.). Continuous data were expressed as the mean ± standard deviation. Categorical variables are presented as counts (and percentages). Comparison of the continuous variables was performed by a two-sample Student’s t-test for normally distributed data. The chi-square test was used to compare the frequency of occurrence.

A multivariable regression model to predict ACI was developed considering 5 CEUS variables (P_TIC_, M_TIC_, P_FC_, S_FC_, and AUC_FC_) and 15 clinical variables (age, gender, BMI, SBP, DBP, history of hypertension, history of diabetes mellitus, FPG, TC, TC, LDL, HDL, leukocytes, lymphocytes, and neutrophils). These CEUS and clinical variables were entered into a backward logistic regression analysis and were chosen from the set of candidates by backward elimination. We also used a classification and regression tree analysis model, which created an inverted tree based on binary splitting choosing a variable value that best separated those with vulnerable plaques from those with stable plaques. Accuracy statistics were computed to assess the model performance including sensitivity, specificity, accuracy, and the area under the receiver operating characteristic curve (ROC).

Pearson’s correlation tests were performed to evaluate the correlation between the parameters, and scatter plots were examined to assess the association between P_TIC_, AUC_FC_, lymphocytes, and neutrophils.

The reproducibility of the parameters was assessed in 20 randomly selected patients whose P_TIC_ and M_TIC_ values were both measured independently by two physicians and twice by the same physician, respectively. The repeatability evaluation adopted a linear correlation analysis and Bland–Altman plots. Bland–Altman plots were used to assess inter-observer variability. Statistical significance was considered to exist at *P* < 0.05.

## Results

### Clinical characteristics

A total of 116 patients were included in the study: 62 symptomatic patients (48 males) with large artery atherosclerosis cerebral infarction and 54 asymptomatic patients (42 males) matched by age and sex. Patient clinical characteristics were summarized in Table 1. The mean values of the leukocyte and neutrophil counts in the symptomatic group were significantly higher than in the asymptomatic group; however, the mean value of the lymphocyte count was significantly lower than that in the asymptomatic group (*P*<0.05).

**Table 1:** Comparison of clinical characteristics between the two groups

Item	Symptomatic patients (*N* = 62)	Asymptomatic patients (*N* = 54)	*t*-value	*P*-value
Gender (F/M) (χ^2^)	14/48	12/42	0.974	1.000
Age (year)	67.7 ± 8.8	64.7 ± 6.8	1.571	0.121
Height (cm)	166.9 ± 6.4	166.6 ± 6.6	0.202	0.840
Weight (kg)	64.0 ± 11.1	64.7 ± 10.6	−0.247	0.805
Body mass index (kg/m^2^)	22.8 ± 3.1	23.2 ± 2.8	−0.681	0.497
SBP (mm Hg)	137.5 ± 15.2	135.3 ± 17. 6	0.495	0.622
DBP (mm Hg)	85.8 ± 9.3	85.7 ± 10.9	0.040	0.968
Diabetes mellitus type 2 (*n*)	24.0	18.0	0.671	0.786
Hypertension (n)	28.0	22.0	0.735	0.795
Fasting plasma glucose (m mol / L)	6.1 ± 1.6	5.7 ± 1.4	0.838	0.407
Total cholesterol (m mol / L)	4.6 ± 1.2	4.5 ± 0.9	0.340	0.736
LDL cholesterol (m mol / L)	2.9 ± 1.1	3.0 ± 0.8	−0.397	0.693
Triglycerides (m mol / L)	1.7 ± 1.3	1.3 ± 0. 8	1.061	0.295
Leukocytes (× 10^9^/L)	7.05 ± 2.33	6.01 ± 1.82	2.235	0.028
Lymphocytes (×10^9^/L)	1.67 ± 0.54	1.99 ± 0.91	−1.929	0.047
Neutrophils (×10^9^/L)	4.59 ± 1.72	3.71 ± 1.59	2.409	0.018

Values are means (±SD). *DM* diabetes mellitus, *BMI* body mass index, *SBP and DBP* systolic and diastolic blood pressures, respectively, *LDL* low-density lipoprotein, *HDL* high-density lipoprotein, *n* number, 1 mmHg = 0.133 kPa

### Comparison of carotid plaque CEUS quantitative parameters

A total of 491 plaques were identified in the 116 patients. Two hundred forty-one plaques were detected in the common carotid artery, and 205 plaques were detected in the internal carotid artery. Though forty-five plaques were detected in the external carotid artery, they were not included in this study because the plaques in this site were not related to cerebrovascular events. Eighty-three patients had multiple plaques (mean, 3.6; range, 1–12), and sixty-six patients had plaques coexisting in the common carotid artery and internal carotid artery. The plaque characteristics of patients with and without history of the previous stroke were similar.

**Fig. 1:**
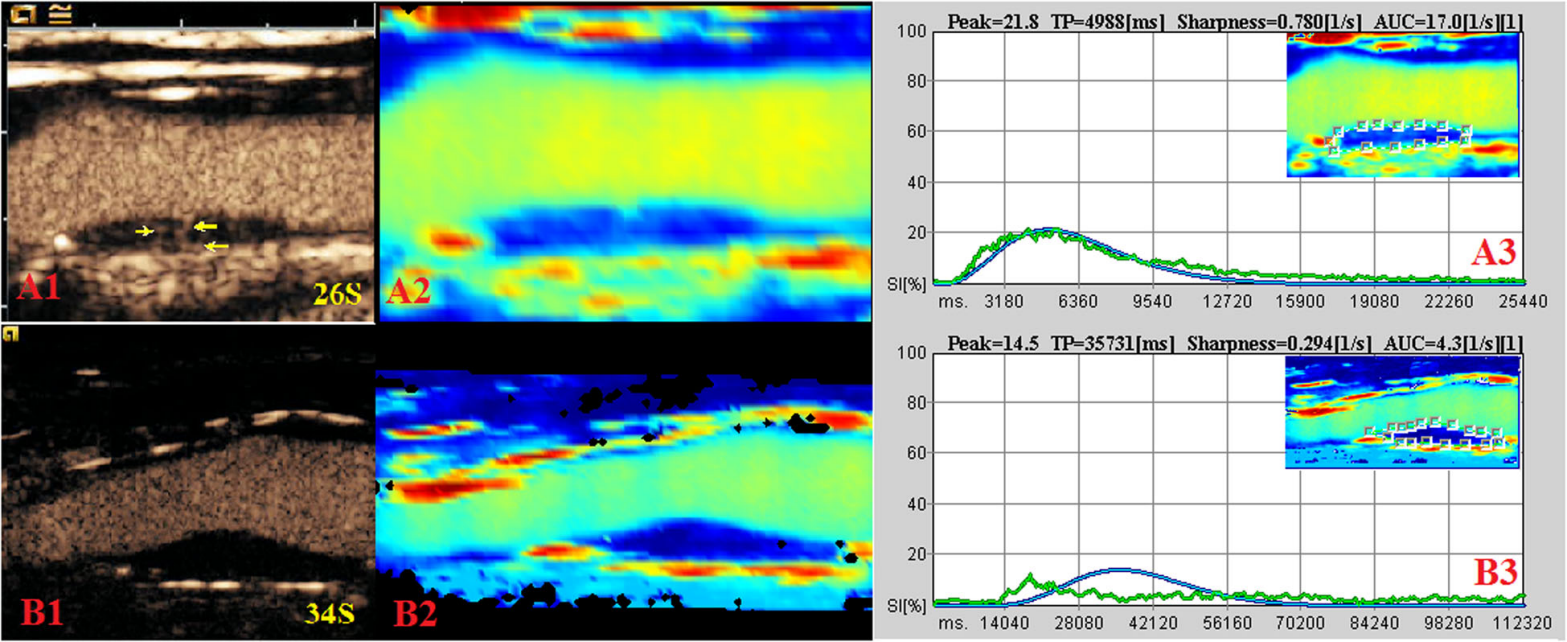
CEUS and quantitative analysis images of carotid atherosclerotic plaques. A1–A3: acute cerebral infarction patients, B1–B3: asymptomatic patients. A1 and B1: the CEUS image shows no enhancement in the carotid plaque with asymptomatic patients, while significant enhancement (arrow) is observed in the carotid plaques with ACI patients. A2 and B2: 3D imaging of the perfusion parameters (parametric maps) is obtained from the carotid plaques using a scale of colors varying from red (maximum signal intensity) to blue (minimum signal intensity), passing through yellow and green. A3 and B3: quantitative analysis images of carotid atherosclerotic plaques; yellow arrow: IPN; green curve: time-intensity curve (TIC), blue curve: time-intensity fitting curve (FC). Numeric values of the peak, TP, sharpness, and AUC were automatically calculated based on the time-intensity curve and are shown at the top of the graphs

An obvious plaque was successfully selected to perform CEUS examination in each subject according to the following selection criteria: those located at the far wall of carotid bifurcation or the initial part of the internal carotid artery; those thicker than 2 mm, choosing the largest; no calcification or the least calcified plaque; and cerebral infraction ipsilateral to the side of the carotid plaque (Fig. 1). The mean values of P_TIC_, M_TIC_, P_FC_, S_FC_, and AUC_FC_ in these cases were significantly higher than those in the controls (all *P*<0.05) (Table 2).

**Table 2:** Comparison of CEUS parameters of carotid plaques between the two groups

Item	Symptomatic patients (N = 62)	Asymptomatic patients (N = 54)	*t*-value	*P*-value
P_TIC_ (dB)	55.08 ± 14.57	42.92 ± 14.63	9.628	<0.001
M_TIC_ (dB)	25.29 ± 8.89	21.88 ± 8.15	1.986	0.046
P_FC_	25.24 ± 8.92	23.89 ± 8.09	1.995	0.041
S_FC_ (1/s)	0.71 ± 0.27	0.20 ± 0.11	8.489	<0.001
AUC_FC_ (1/s)	17.22 ± 8.38	4.40 ± 1.97	7.292	<0.001

P_TIC_ peak of time-intensity curve, *M*_*TIC*_ mean of time-intensity curve, *P*_*FC*_ peak of fitting curve, *S*_*FC*_ sharpness of fitting curve, *AUC*_*FC*_ area under the fitting curve; n, number

### Comparison of data from symptomatic patients with different histories in two subgroups

To prove the correlation between IPN and peripheral leukocytes in Subgroups 1 and 2, a stratified analysis was used. There were no significant differences between Subgroups 1 and 2 in CEUS or leukocyte count. The neutrophil count showed a decreasing tendency in Subgroup 1, while P_TIC_ and AUC_FC_ showed an increasing trend in Subgroup 2. However, this difference was not statistically significant (Table 3).

**Table 3:** Comparison of data from the symptomatic patients with different histories in two subgroups

Item	Subgroup 1 (*N* = 28)	Subgroup 2 (*N* = 34)	*t*-value	*P*-value
Leukocytes (×10^9^/L)	6.95 ± 2.35	7.13 ± 2.31	−1.671	0.100
Lymphocytes (×10^9^/L)	1.73 ± 0.59	1.62 ± 0.50	1.029	0.197
Neutrophils (×10^9^/L)	4.43 ± 1.62	4.72 ± 1.80	−1.812	0.073
P_TIC_ (dB)	57.38 ± 15.22	53.19 ± 14.03	1.928	0.052
M_TIC_ (dB)	27.33 ± 9.01	23.61 ± 8.79	1.848	0.068
P_FC_	26.54 ± 9.12	24.17 ± 8.76	0.827	0.257
S_FC_ (1/s)	0.69 ± 0.30	0.73 ± 0.25	−0.563	0.344
AUC_FC_ (1/s)	18.32 ± 9.01	16.31 ± 7.86	1.857	0.057

*P*_*TIC*_ peak of time-intensity curve, *M*_*TIC*_ mean of time-intensity curve, *P*_*FC*_ peak of fitting curve, *S*_*FC*_ sharpness of fitting curve, *AUC*_*FC*_ area under the fitting curve, *n* number

### Repeatability test

**Fig. 2:**
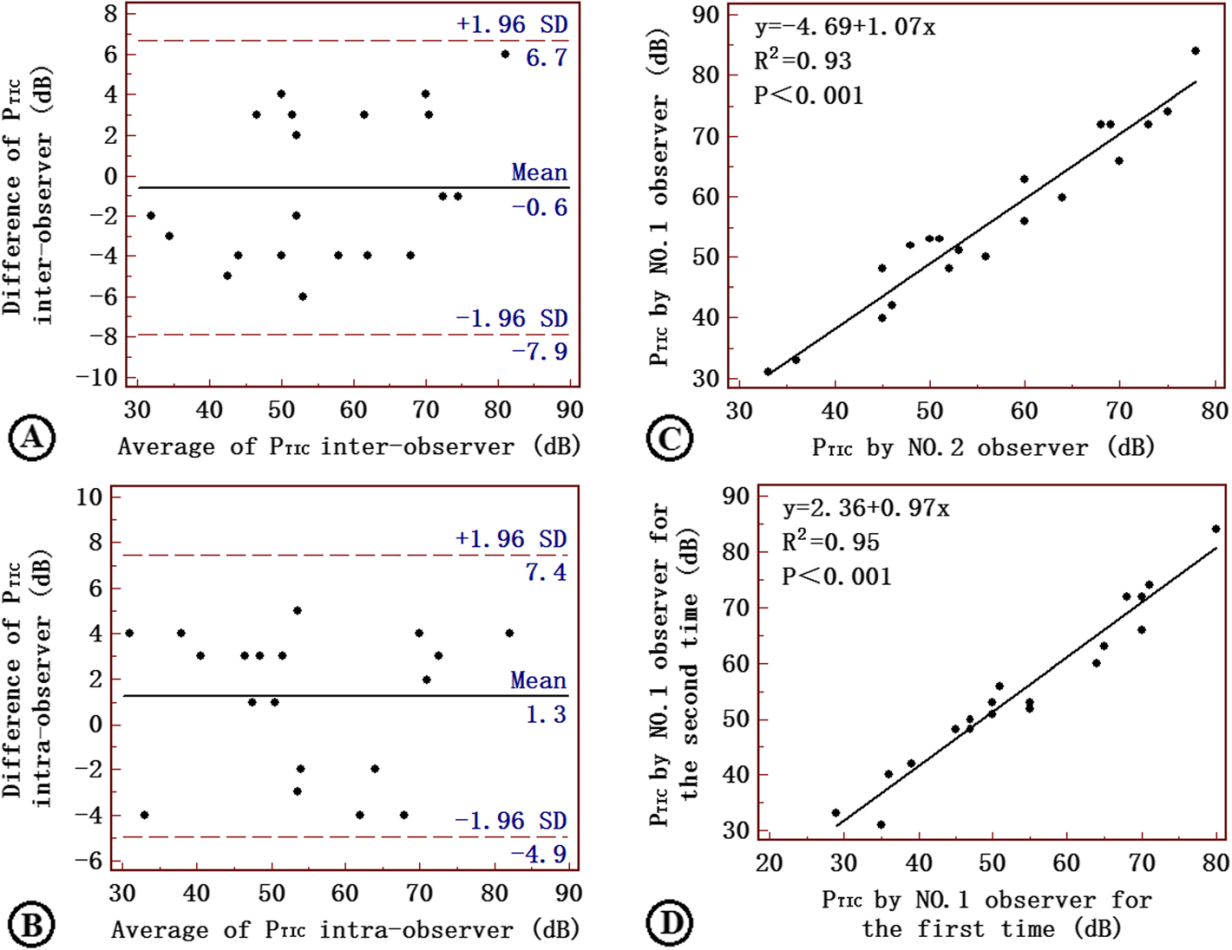
Repeatability was analyzed by Bland–Altman Plots (**a**-**b**) and linear correlation analysis (**c**-**d**) in the inter- and intragroups. The results showed a consistent trend in the difference value and the mean value of P_TIC_ by repeated measurement. Inter- and intragroup comparisons had a high degree of consistency. P_TIC_: peak of the time-intensity curve

The repeatability test showed that intergroup and intragroup comparisons had a high degree of consistency (Fig. 2).

### Correlations between carotid CEUS parameters and peripheral blood leukocyte count

**Fig. 3:**
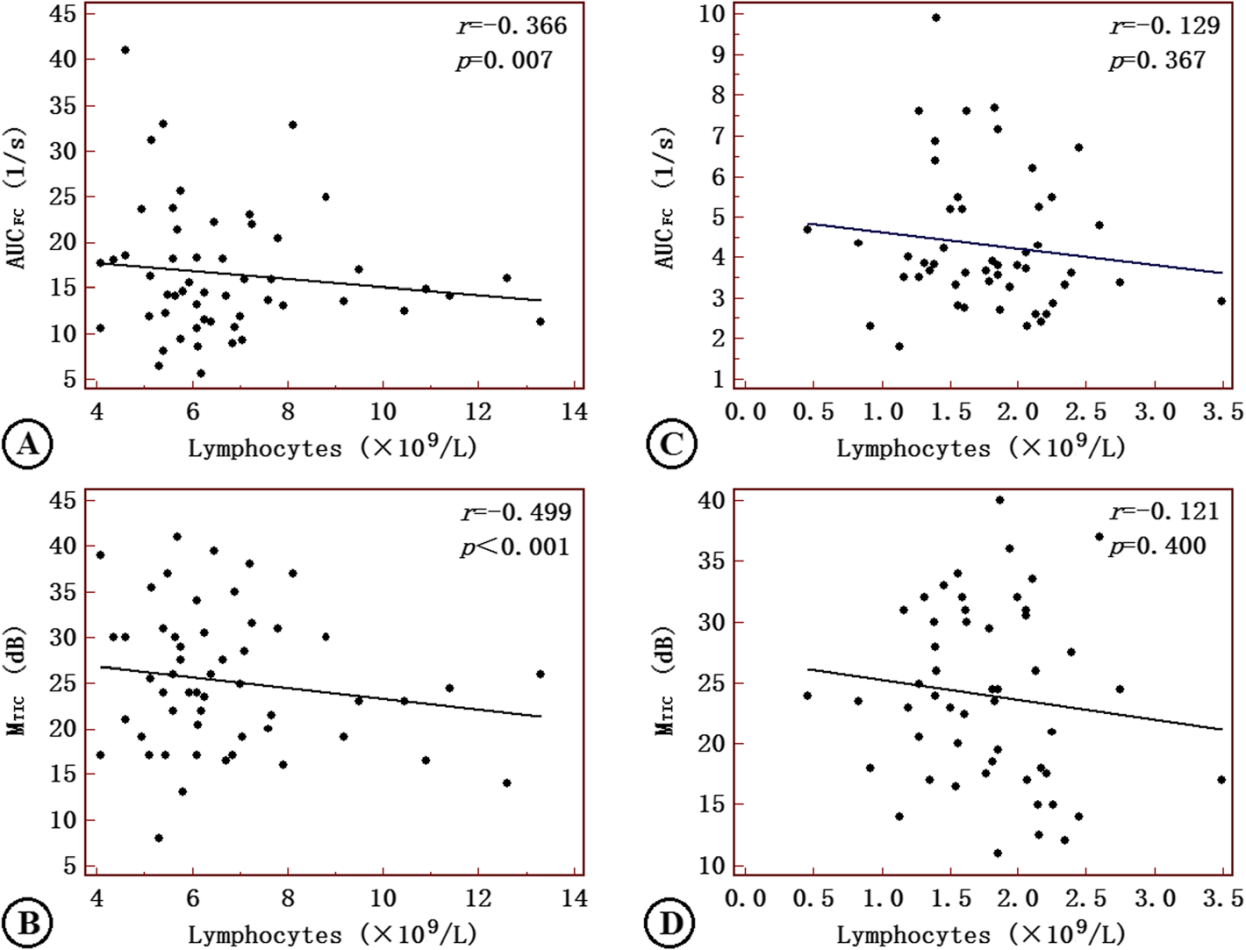
Correlations of AUC_FC_ and M_TIC_ with lymphocytes in ACI patients (**a**-**b**) and asymptomatic patients (**c**-**d**). AUC_FC_: area under the fitting curve; M_TIC_: mean of the time-intensity curve; AIS: acute ischemic stroke. Correlation coefficients and *P*-values are given in the graphs

**Fig. 4:**
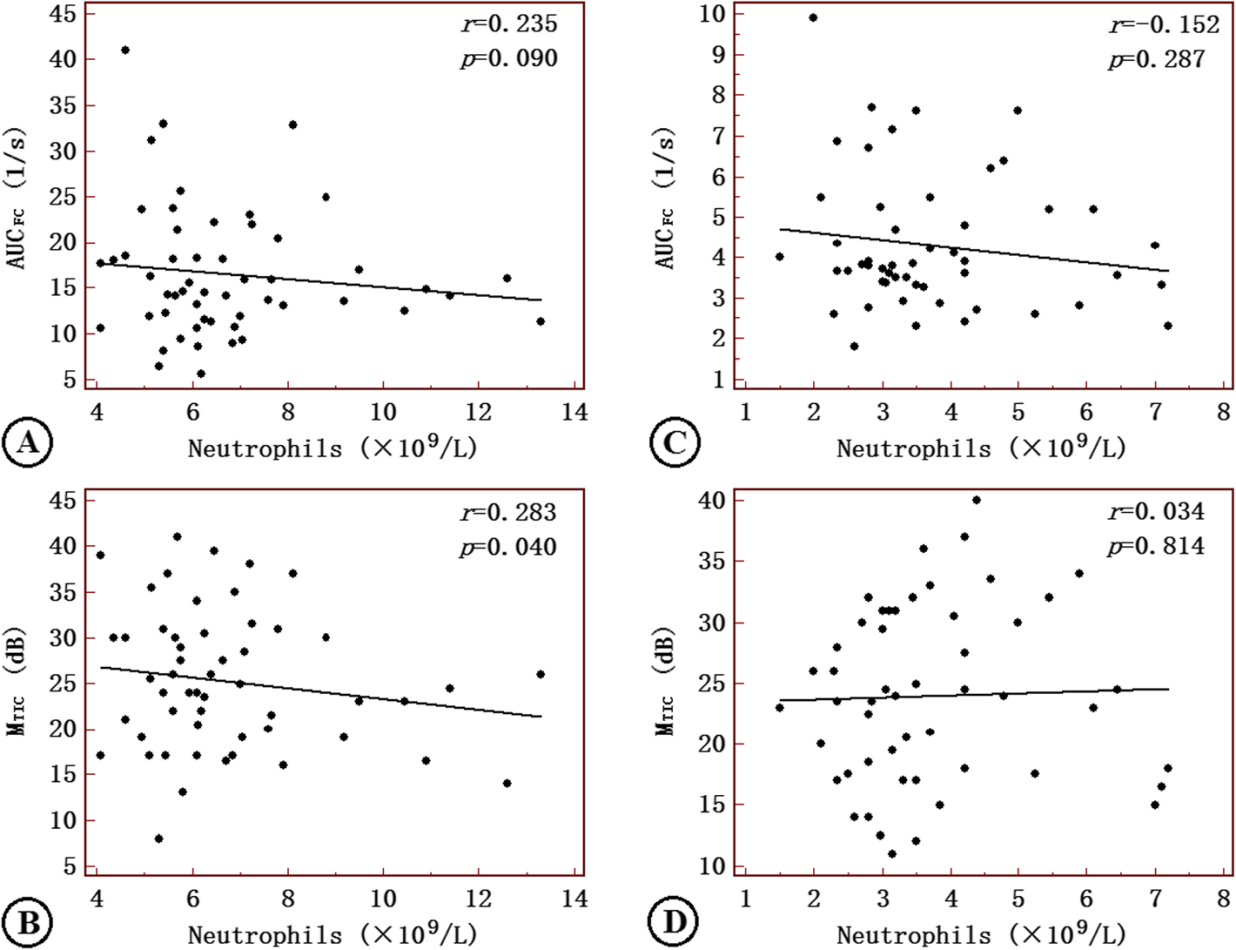
Correlations of AUC_FC_ and M_TIC_ with neutrophils in ACI patients (**a**-**b**) and asymptomatic patients (**c**-**d**). AUC_FC_: area under the fitting curve; M_TIC_: mean of the time-intensity curve; AIS: acute ischemic stroke. Correlation coefficients and *P*-values are given in the graphs

M_TIC_ and AUC_FC_ modestly correlated with lymphocytes in the symptomatic patients (Fig. 3), while M_TIC_ correlated closely with neutrophils only in the symptomatic group (Fig. 4).

### Diagnostic accuracy

Using a backward logistic regression model, only 2 variables (AUC_FC_ and M_TIC_) were considered to be simultaneously significant and remained independent predictors of ACI when evaluated against other clinical and CEUS parameters.

**Fig. 5:**
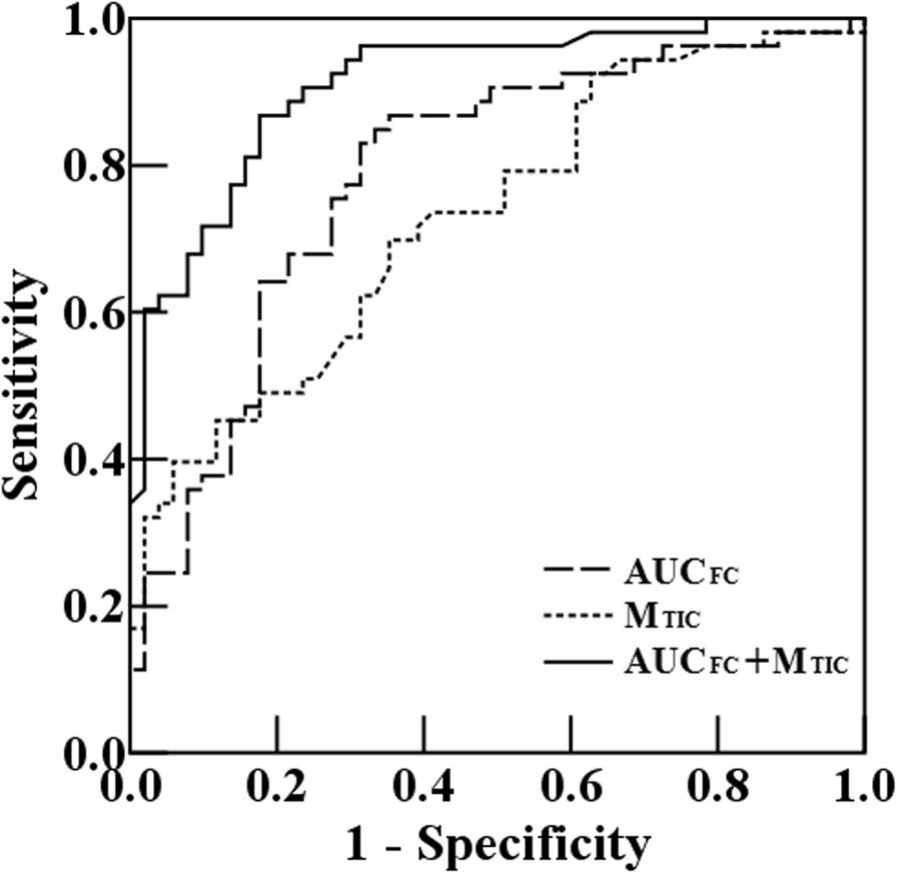
Receiver operating characteristic (ROC) curve for the CEUS parameters’ ability to predict vulnerable plaques in patients with ACI

ROC analysis showed the areas under the curves of AUC_FC_ and M_TIC_ to be 0.787 and 0.729, respectively (Fig. 5). The optimal AUC_FC_ and M_TIC_ cutoff values for detecting ACI were 9.57 and 20.8 dB with 83.0 and 69.8% sensitivity, 68.6 and 64.7% specificity, and 75.8 and 67.3% accuracy, respectively (Table 3).

Subsequently, a further classification tree model using a binary recursive partition method also confirmed significant contributions from the same 2 variables (AUC_FC_ and M_TIC_), resulting in an improvement in overall diagnostic accuracy that was superior to that for each measure alone (Table 4).

**Table 4:** Cutoff, Sensitivity, Specificity, and Accuracy for AUC_FC_ and M_TIC_

Variable	Cutoff	Sensitivity, %	Specificity, %	Accuracy, %	AUROC (95% CI)
AUC_FC_ (1/s)	9.57	83.0	68.6	75.8	0.787 (0.696, 0.861)
M_TIC_ (dB)	20.8	69.8	64.7	67.3	0.729 (0.633, 0.811)
AUC_FC_ + M_TIC_	8.8 + 20.8	87.1	81.5	84.3	0.911 (0.839, 0.958)

*AUC*_*FC*_ area under the fitting curve, *M*_*TIC*_ mean of time-intensity curve, *CI* confidence interval, *AUROC* area under the ROC curve, *CI* confidence interval

Using AUC_FC_ at a cutoff of 8.8 and M_TIC_ at a cutoff of 20.8, we were able to correctly identify 54 of 62 ACI patients (87.1%) and 44 of 54 non-ACI patients (81.5%) with an overall accuracy of 84.3%. The area under the ROC curve was 0.911.

## Discussions

Cerebral infarction has some of the highest morbidity and mortality rates worldwide. Atherosclerotic plaque rupture is the leading cause of ACI [16]. Therefore, exploring a safe and accurate diagnostic method to identify vulnerable plaques will contribute to decreasing cardio-cerebrovascular events [17]. A previous study showed that CEUS could identify vulnerable plaques by evaluating IPN (and plaque ulceration) [18]. The leukocytes in circulation can colonize plaques and then induce an inflammatory response that stimulates the formation of neovascularizations in atherosclerotic plaques [19]. The present study evaluated IPN in obvious plaques using CEUS and analyzed the correlations between CEUS quantitative parameters and differential leucocyte counts. The present study also revisited the diagnostic value of CEUS in identifying vulnerable atherosclerotic plaques.

The quantitative parameters of CEUS (P_TIC_, M_TIC_, P_FC_, S_FC_, and AUC_FC_) reflect the microvessel density of vulnerable atherosclerotic plaques and their blood perfusion and perfusion patterns from different sides. This study showed that the mean values of P_TIC_, M_TIC_, P_FC_, S_FC_, and AUC_FC_ in the ACI group were significantly higher than those in the asymptomatic group. This indicated that the carotid atherosclerotic plaques of patients with cerebral infarction had more neovascularization and vulnerability. Moguillansky et al. [20] established an artery atherosclerotic plaque model. They performed atherosclerotic plaque CEUS and compared it with pathological results. They found that the quantitative parameters of CEUS were closely related to the microvessel density of the plaque and suggested that CEUS could evaluate its pathological features and stability. The IPN, the second- or third-order branches of the vasa vasorum, primarily come from the adventitial vasa vasorum, which twists through medial smooth muscle to the intima [21]. Thus, the partial oxygen pressure in the IPN is the lowest. Previous research has shown that increased intima-media thickness and the formation of atherosclerotic plaques could further decrease the partial oxygen pressure of the terminal vasa vasorum and form a hypoxic-ischemic micro-environment, which stimulates neovascularization under the intima or within plaques [22]. These neovascularizations, which are immature and lack smooth muscle cells and a complete basement membrane in the wall with a larger interstitial space between endothelial cells, are thus prone to rupture and hemorrhage, resulting in cardio-cerebrovascular events [23]. Research by Hosseini et al. [24] of 179 symptomatic patients with carotid artery stenosis of more than 50% reported that the incidence of IPN and hemorrhage was 63.7% and the recurrence of ipsilateral cerebral ischemic events was up to 92%. A similar study showed that intraplaque hemorrhage occurred in 57% of specimens from symptomatic patients with internal carotid artery stenosis who had undergone an internal carotid endarterectomy. The study also found that IPN grades classified by CEUS have a direct correlation with neo-angiogenesis density in the specimen and are closely related to intraplaque hemorrhage and the percentage of macrophages [25]. This indicates that carotid artery CEUS could estimate intraplaque hemorrhage and reflect inflammatory activities in the plaque by measuring neoangiogenesis [26].

Atherosclerosis, vascular inflammation, and neovascularization are closely related [27]. There are two hypotheses for atherosclerotic vascular inflammation and intraplaque inflammatory activities. According to the traditional concept, vascular inflammation is considered to be an “inside-out” response focused on monocyte adhesion and lipid oxidation hypotheses [28]. However, increasing evidence supports an “outside-in” hypothesis, in which intraplaque inflammation is initiated in the arterial adventitia and progresses inward toward the intima [29]. The intraplaque inflammatory activities and neovascularization all stem from a continuous vascular inflammatory reaction. This hypothesis reminds us that an inflammatory state of circulating blood may spread into atherosclerotic plaques through abundant and immature neo-angiogenesis [30]. This study found that the perivascular total leukocyte and neutrophil counts of patients with ACI were significantly higher but that the lymphocyte count was significantly lower. An increased neutrophil count is positively correlated with M_TIC_, which reflects the IPN. A decreased lymphocyte count is negatively correlated with M_TIC_ and AUC_FC_, reflecting intraplaque blood perfusion. Asuman et al. [31] demonstrated that the neutrophil to lymphocyte ratio (N/L ratio) was highest in patients with ACI compared with transient cerebral ischemia and control subjects. Inflammation plays a fundamental role in the development and progression of atherosclerosis, and leukocytes participate in the plaque formation and destabilization, thereby inducing acute thrombotic events. Therefore, the variation in the perivascular leucocyte could be a novel noninvasive marker for cerebrovascular events [32]. Besides, a clinical study demonstrated that with intraplaque hypermetabolism, high-oxygen consumption macrophages more easily produce a hypoxic microenvironment compared with increased intima-media thickness [33]. Oxygen diffusive disorder in thickened intima-media and increased oxygen consumption of inflammatory cells both lead to a hypoxic microenvironment state and stimulate neovascularization, intraplaque hemorrhage, and the rupture of atherosclerotic plaques [7]. A recent study showed that the adventitia not only provides structural support for the vessel walls but also facilitates atherosclerosis, as well as the formation of neo-intima and vulnerable plaques by “outside-in” inflammatory cells and the transfer of fibroblasts [34]. Though prospective observational data is still lacking, more advanced modalities have been introduced, including intravascular ultrasound (IVUS), virtual-histology IVUS, optical coherence tomography (OCT), et al. They can better delineate microstructures of plaques, and may potentially lead to a major shift in the management of millions of patients with ACI.

This study has some limitations. First, the number of patients enrolled was relatively small, and we did not compare the differences between the plaque ulcer group and the plaque integrity group. Second, accuracy may have been affected by evaluating intraplaque inflammatory activities through the peri-vascular leukocyte count because it is not equivalent to the inflammatory cell count in the plaques.

## Conclusions

In conclusion, carotid artery CEUS could be used to assess plaque vulnerability by quantitatively analyzing IPN. Carotid artery CEUS could also serve as a visualization diagnostic tool for the adventitia vasa vasorum. This may provide a new perspective on the “outside-in” theory of inflammatory activities in atherosclerotic plaques.

## Abbreviations

ACI: Acute Cerebral Infarction
AUC_FC_: Area Under the Curve
CA: Calcified Area
CEUS: Contrast-enhanced Ultrasound
FC: Fitting the Gamma Curve
IPN: Intraplaque Neovascularization
M_TIC_: Mean Time-signal Intensity
OPA: Obvious Plaque Area
P_FC_: Maximum Peak Value
P_TIC_: Peak Time-signal Intensity
ROC: Receiver Operating Characteristic Curve
S_FC_: Sharpness of FC
TIC: Time-signal Intensity; Curve
